# Genetic Analysis of Hedgehog Signaling in Ventral Body Wall Development and the Onset of Omphalocele Formation

**DOI:** 10.1371/journal.pone.0016260

**Published:** 2011-01-20

**Authors:** Daisuke Matsumaru, Ryuma Haraguchi, Shinichi Miyagawa, Jun Motoyama, Naomi Nakagata, Frits Meijlink, Gen Yamada

**Affiliations:** 1 Global COE "Cell Fate Regulation Research and Education Unit", Department of Organ Formation, Institute of Molecular Embryology and Genetics (IMEG), Kumamoto University, Kumamoto, Japan; 2 Department of Medical Life Systems, Doshisha University, Kyoto, Japan; 3 Center for Animal Resources and Development (CARD), Kumamoto University, Kumamoto, Japan; 4 Hubrecht Institute, KNAW and University Medical Center, Utrecht, The Netherlands; The University of Hong Kong, China

## Abstract

**Background:**

An omphalocele is one of the major ventral body wall malformations and is characterized by abnormally herniated viscera from the body trunk. It has been frequently found to be associated with other structural malformations, such as genitourinary malformations and digit abnormalities. In spite of its clinical importance, the etiology of omphalocele formation is still controversial. Hedgehog (Hh) signaling is one of the essential growth factor signaling pathways involved in the formation of the limbs and urogenital system. However, the relationship between Hh signaling and ventral body wall formation remains unclear.

**Methodology/Principal Findings:**

To gain insight into the roles of Hh signaling in ventral body wall formation and its malformation, we analyzed phenotypes of mouse mutants of *Sonic hedgehog* (*Shh*), *GLI-Kruppel family member 3* (*Gli3*) and *Aristaless-like homeobox 4* (*Alx4*). Introduction of additional *Alx4^Lst^* mutations into the *Gli3^Xt/Xt^* background resulted in various degrees of severe omphalocele and pubic diastasis. In addition, loss of a single *Shh* allele restored the omphalocele and pubic symphysis of *Gli3^Xt/+^; Alx4^Lst/Lst^* embryos. We also observed ectopic Hh activity in the ventral body wall region of *Gli3^Xt/Xt^* embryos. Moreover, tamoxifen-inducible gain-of-function experiments to induce ectopic Hh signaling revealed Hh signal dose-dependent formation of omphaloceles.

**Conclusions/Significance:**

We suggest that one of the possible causes of omphalocele and pubic diastasis is ectopically-induced Hh signaling. To our knowledge, this would be the first demonstration of the involvement of Hh signaling in ventral body wall malformation and the genetic rescue of omphalocele phenotypes.

## Introduction

The embryonic visceral organs transiently protrude out of the body trunk during mid-gestation, where they are covered with the peritoneal membrane. Subsequently they return to the peritoneal cavity in both mouse and human embryos. This transient embryonic hernia of the viscera is termed the physiological umbilical hernia [1], [2]. According to previous reports, protrusion of the midgut loop through the umbilical ring is due to the rapid expansion in the volume of visceral organs, exceeding the space of the peritoneal cavity [1], [3]. However, the molecular mechanisms underlying the ventral body wall formation, including physiological umbilical herniation, are still unclear.

An omphalocele is a major ventral body wall malformation characterized by a severe umbilical defect with herniation of visceral organs covered with peritoneum and amnion [2], [4], [5]. The frequency is reported to be approximately 1 in 4,000 live births [6]–[8]. In spite of its high incidence, the cause of omphalocele is controversial; it might be due to the failure of recovery of the physiological umbilical hernia or to a midline defect at the transition zone between the ectoderm and mesoderm [7], [9]–[12]. Omphaloceles are frequently associated with other structural malformations such as cardiac, anorectal and digit malformations in more than 50% of cases [5], [13], [14]. For instance, patients with omphalocele-exstrophy-imperforate anus-spinal defects complex (OEIS complex, OMIM: 25840) or bladder exstrophy (OMIM: %600057) exhibit defects not only in the body wall region but also in urogenital organs and its adjacent tissues, including the pelvic girdle [15]–[18]. Our understanding of these malformations is hampered by the complexity of these syndromes. Even the nomenclature and definitions for syndromic congenital malformations are still controversial [19]–[21].

Several genetically-modified animals have been reported to display abnormalities in the body wall region. Such reports include cases of mutants of *Msh-like homeobox 1* and *2* (*Msx1/2*), *Transcription factor AP-2 alpha* (*Tcfap2α*), *Paired-like homeodomain transcription factor 2* (*Pitx2*), *Insulin-like growth factor 2* (*Igf2*), *Igf2 receptor* (*Igf2r*), *Transforming growth factor beta 2* and *3* (*Tgfβ2/3*), *Bone morphogenetic protein 4* (*Bmp4*) and *Bmp receptor type Ia* (*BmprIa*) [3], [22]-[33]. Of note, most of these animals had accompanying limb deformities.

The Hedgehog (Hh) signaling pathway is an essential growth factor signaling pathway involved in many developmental contexts, including digit formation. One of the Hh ligands, Sonic hedgehog (Shh), is secreted from the posterior mesenchymal region of limb buds, the zone of polarizing activity. It is suggested that the anterior-posterior Shh gradient, together with a temporal gradient of exposure to Shh signaling, may specify digit number and identity [34]–[39]. Previous studies suggested that digit abnormalities such as polydactyly frequently accompany ectopic Hh signal induction in anterior limb buds [40], [41]. Both inactivation of *Patched 1* (*Ptc1*, a Hh signal repressor gene) and constitutive activation of *Smoothened* (*Smo*, a Hh signal transducer gene) or *GLI-Kruppel family member 2* (*Gli2,* a Hh signal transcription factor gene) resulted in polydactylous phenotypes [42]–[45]. As for the mutants with body wall phenotypes, *BmprIa*, *Msx1/2* and *Tcfap2α* mutants exhibited ectopic expression of *Shh* gene or its signaling genes (*Gli1* or *Ptc1*) in their limb buds or other tissues [46]–[48]. Of note, the mutants of *GLI-Kruppel family member 3* (*Gli3*) or *Aristaless-like homeobox 4* (*Alx4*) also displayed body wall abnormalities, polydactylies and ectopic Hh signal activity in limb buds [40], [41], [49]–[58]. However, the correlation between omphalocele formation and Hh signaling has not yet been examined.

In this study, we investigated the participation of Hh signaling in ventral body wall formation and the pathogenic mechanisms leading to its malformation by utilizing a series of genetically-modified mouse systems. The phenotypic coordination of ventral body wall, digit and pelvic girdle formations is also discussed. We analyzed the lower body wall phenotypes of combinatorial mutants of *Shh*, *Gli3* and *Alx4* genes. We also analyzed conditional gain-of-function mutants of Hh signaling and revealed the Hh signal dose-dependent pathogenesis of omphalocele and pubic diastasis phenotypes. These results suggest that Hh signaling regulates omphalocele formation and shed light on the pathogenic mechanisms underlying a broad spectrum of lower body malformations.

## Materials and Methods

### Mouse strains and embryos

The mutant mice used herein were *Shh* [59], *Gli3^Xt^* (*Xt^J^*) [50], *Alx4^Lst^* (*Lst^J^*) [54], [55], *Gli1-CreER^T2^* [60], *Shh-CreER^T2^* [61], *Rosa26R* [62], *CAGGS-CreER™* [63], *Rosa26-SmoM2* [64] and *del5-LacZ reporter* [65], [66]. The genotypes of each strain were determined as reported previously. To obtain *Gli3^Xt^; Alx4^Lst^; Shh* compound mutant embryos, single, double or triple heterozygous male and female mice were crossed. Noon of the day when the vaginal plug appeared was designated as embryonic day 0.5 (E0.5). Embryos for each experiment were collected from more than three independent pregnant females. All experimental procedures and protocols for animal studies were approved by the Committee on Animal Research of Kumamoto University (B22-198, B22-200, B22-201 and B22-202).

### Preparation of tamoxifen

The tamoxifen (TM)-inducible Cre recombinase system removes the floxed sequence of the target genome [67]–[69]. TM (Sigma, St. Louis, MO, USA) was dissolved in sesame oil (Kanto chemical, Tokyo, Japan) at a final concentration of 10 mg/ml [65], [70], [71].

### Hh-responded cell contribution analysis

To analyze the cell contribution that responded to Hh signaling, we utilized the *Gli1-CreER^T2^; Rosa26R* system [60]. The *Gli1-CreER^T2^* mice were crossed with *Rosa26R* Cre-indicator (*R26R*) mice to obtain *Gli1-CreER^T2^/+; R26R/R26R* males, which were subsequently crossed with ICR females [60], [72]. Time-mated ICR females were administered TM (2 mg per 40 g maternal body weight (bw)) orally with a gavage needle. Mouse embryos were processed for whole-mount X-gal staining.

### Hh signal gain-of-function experiments

For gain-of-function experiments of Hh signaling, the *Rosa26-SmoM2* (*R26-SmoM2*) homozygous female mice were crossed with the Cre-driver mice, such as *CAGGS-CreER™* transgenic male mice [63], [64], [73]. The pregnant *R26-SmoM2* females were treated with TM (1 mg, 2 mg or 4 mg per 40 g bw) orally with a gavage needle. Embryos were collected, and their morphology was investigated between mid-gestation and perinatal stages. No overt teratological effects were observed in wild-type embryos after TM administration under these conditions [65], [70], [71].

### Histological analyses

Mouse embryos were fixed overnight in 4% paraformaldehyde (PFA) (Sigma) with PBS, dehydrated through methanol, embedded in paraffin, and 8 µm serial sections were prepared. Hematoxylin and Eosin (HE) staining and X-gal staining were processed by standard procedures [65], [74], [75]. For skeletal staining, dehydrated embryos were skinned, eviscerated and refixed in 95% ethanol for several days. Cartilage staining was performed for two days by incubation in 0.03% Alcian blue 8GX (Sigma), dissolved in 80% ethanol/20% acetic acid. After washing the embryos in 95% ethanol for five days, they were stained with 0.0025% Alizarin red S (Sigma) in 1% KOH for two days. Subsequently, they were treated with 1% KOH for 6 hours. Finally, the embryos were cleared with 20%, 40% and 60% glycerol and stored in 60% glycerol.

### Statistical analysis

For the statistical analyses of the length of an extra digit, the length was measured with a slide gauge. Data were analyzed using a Student's *t*-test (two tailed). A probability of less than 0.001 was considered to indicate statistical significance. Values are given as the means±SD.

### *In situ* hybridization for gene expression analysis

*In situ* hybridization was performed on PFA-fixed and dehydrated embryos. Samples were rehydrated, and incubated in 6% hydrogen peroxide solution for 1 hour. After washing in PBS containing 0.1% Tween 20, samples were incubated in 1 µg/ml ProteinaseK for 18 minutes, and refixed with fixing solution (4% PFA/0.2% glutaraldehyde) for 10 minutes. After washing with PBS containing 0.1% Tween 20, overnight incubation was performed in a buffer (50% formamide, 5x saline sodium citrate, 50 µg/ml yeast tRNA, 1% sodium dodecyl sulfate, 50 µg/ml heparin) at 65°C. Subsequent overnight hybridization was performed in a buffer with 0.5 µg/ml riboprobes at 65°C. Samples were washed in 50% formamide, 5x saline sodium citrate, 1% sodium dodecyl sulfate and 50% formamide, 2x saline sodium citrate for each 1 hour at 65°C, then 140 mM NaCl, 2.7 mM KCl, 0.1% Tween 20, 25 mM Tris-HCl (pH 7.5) for 5 minutes at room temperature before incubating with blocking solution (25% heated FBS in 140 mM NaCl, 2.7 mM KCl, 25 mM Tris-HCl (pH 7.5), 0.1% Tween 20) for 1 hour. Samples were treated with anti-digoxigenin antibody (Roche, Mannheim, Germany) in a blocking solution overnight at 4°C. After washing, samples were equilibrated in 100 mM NaCl, 50 mM MgCl_2_, 0.1% Tween 20, and 100 mM Tris-HCl (pH 9.5) including 2 mM levamisole (Sigma) and incubated in BM purple AP Substrate solution (Roche). *Myogenin* (kindly provided from Dr. Shosei Yoshida) and *Gli1* [65] probes were used. The preparation of the digoxigenin-labeled probes was performed according to the manufacturer's instructions (Roche).

### Cell death analysis

Embryos were collected in PBS, rinsed in PBS and stained with 500 ng/ml Acridine Orange base (Fluka, St. Gallen, Switzerland) for 30 minutes. These procedures were performed at 37°C. Samples were then rinsed briefly in PBS, followed by fluorescence microscopy.

## Results

### Ventral body wall formation and the developmental coordination between the ventral body wall and the pelvic girdle

**Figure 1:**
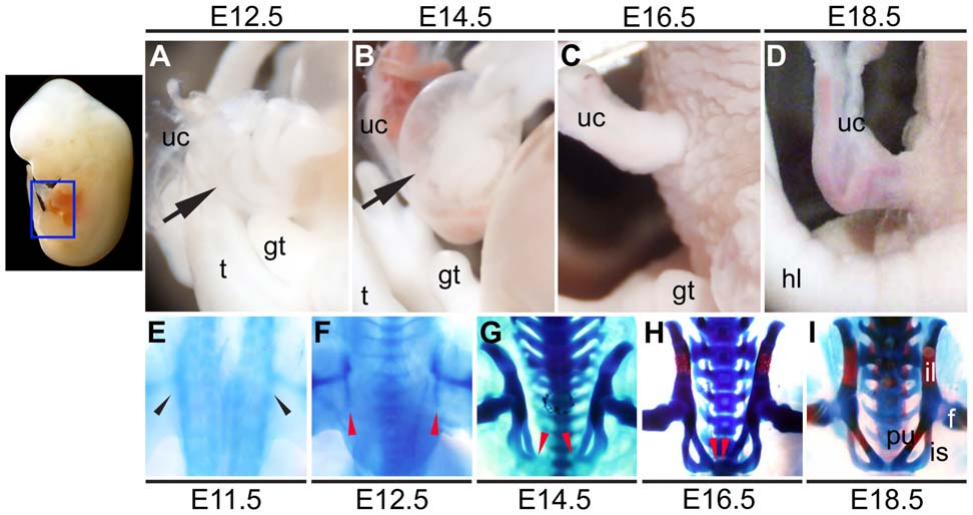
The development of the ventral body wall and the pelvic girdle. Wild-type embryos exhibited a physiological umbilical hernia in the ventral body wall at E12.5 and E14.5 (**A, B**). Black arrows indicate the physiological umbilical hernia. The physiological umbilical hernias were recovered in wild-type late staged embryos at E16.5 and E18.5 (**C, D**). The anlagen of the pelvic girdle start to be observed at around E11.5 (**E**; black arrowheads). The jointing of the hip bones (pubic symphysis) was not formed yet in wild-type embryos at E12.5 and E14.5 (**F, G**). The embryonic pelvic girdle develops to the midline at E16.5 and the pelvic ring is formed at E18.5 (**H, I**). Red arrowheads indicate the midline edges of the pelvic girdle primordia (future symphysial surfaces of the pubis). f: femur, gt: genital tubercle, hl: hind limb, il: iliac bone, is: ischial bone, pu: pubic bone, t: tail, uc: umbilical cord.

We analyzed the development of the embryonic body wall in a series of wild-type murine embryos. The protrusion of embryonic viscera covered with a peritoneal membrane (physiological umbilical hernia) was apparent by E12.5 (Fig. 1A,B) [1], [2]. It was subsequently recovered from E16.5 onwards when the ventral body wall closed (Fig. 1C). As a result, only the umbilical cord could then be observed outside of the ventral body wall (Fig. 1C,D). We also analyzed pelvic girdle morphogenesis because patients with several congenital diseases, such as exstrophy of the cloaca, display malformations not only in the body wall region but also in the urogenital organs and the pelvic girdle [15]–[18]. The bilateral primordia (cartilaginous elements) of the pelvic girdle started to be perceptible from E11.5 (Fig. 1E) [76] and they were positioned in parallel along with the body trunk at E12.5 (Fig. 1F). Subsequently, the edges of the pubic bones started to close, but were not yet connected at the stage of the physiological umbilical hernia (at E14.5) (Fig. 1G). Consistent with the recovery of the physiological umbilical hernia, the pubic symphysis was formed at about E16.5 or later (Fig. 1H,I).

### Genetic interaction between *Gli3* and *Alx4* genes and their involvement in the Hedgehog signaling pathway

**Figure 2:**
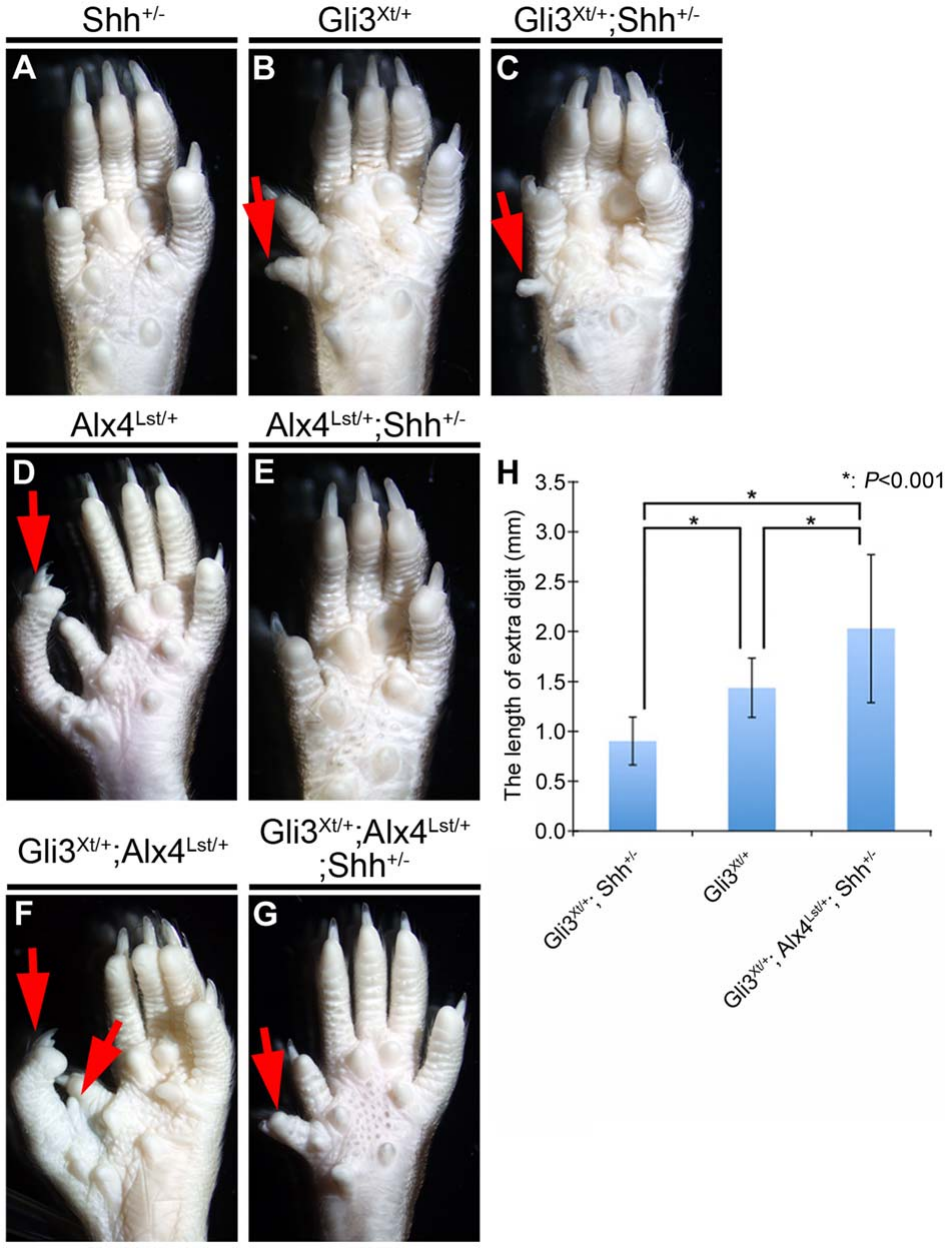
The digit phenotypes of *Shh*, *Alx4* and *Gli3* heterozygotes. *Shh* heterozygous mutants (*Shh^+/−^*) showed a normal number of digits (**A**). Both *Gli3* and *Alx4* heterozygotes (*Gli3^Xt/+^* or *Alx4^Lst/+^*) displayed polydactyly phenotypes in the hind limbs (**B, D**). Polydactylies were partially restored (**C**) or fully restored (**E**) by the addition of a *Shh* heterozygous mutation in *Gli3^Xt/+^* or *Alx4^Lst/+^* heterozygotes. The *Gli3^Xt/+^; Alx4^Lst/+^* double heterozygotes displayed polydactyly with more than two extra digits (**F**). Polydactylies in the *Gli3^Xt/+^; Alx4^Lst/+^* double heterozygotes were partially restored by the additional introduction of a *Shh* heterozygous mutation (*Gli3^Xt/+^; Alx4^Lst/+^; Shh^+/−^*) (**G**). Red arrows indicate extra digits. The length of the extra digit was measured for each genetic combination (**H**). An asterisk indicates statistical significance based on the comparison of each mutant by Student's *t*-test. The results are presented as the means±SD. *P<0.001.

According to previous studies, several human patients and genetically-modified mouse models with body wall phenotypes often have accompanying digit abnormalities [3], [20], [26], [28], [47], [51], [53]. Judging by the causative genes of digit abnormalities, we hypothesized that Hedgehog (Hh) signaling may also be involved in the onset of body wall malformation. To examine this hypothesis, we analyzed combinatorial mutants for Hh and putative Hh signaling related genes: *Shh*, *Gli3* and *Alx4*. Hence, we analyzed the phenotypes of the hind limb, which is a well-analyzed system for examining genetic relationships among developmental genes. Wild-type and *Shh^+/−^* mice displayed normal digit morphology (Fig. 2A). Both *Gli3^Xt/+^* and *Alx4^Lst/+^* single heterozygotes showed preaxial polydactyly (Fig. 2B,D) [40], [49], [54]. The size of the extra digit in *Gli3^Xt/+^; Shh^+/−^* mice was smaller than that of *Gli3^Xt/+^* mice (Fig. 2C). On the other hand, this digit phenotype was completely restored in *Alx4^Lst/+^; Shh^+/−^* mice (Fig. 2E). Moreover, *Gli3^Xt/+^; Alx4^Lst/+^* mice displayed severe polydactyly (two extra digits) (Fig. 2F). This phenotype was also partially restored by the addition of the *Shh* mutation (Fig. 2G). To quantify the effects of the gene mutations, we analyzed the significance of the length of the extra digit (Fig. 2H). The introduction of an additional *Shh* mutation significantly reduced the length of the extra digit (by a comparison between *Gli3^Xt/+^* versus *Gli3^Xt/+^; Shh^+/−^*: 1.44±0.30, n = 30 versus 0.90±0.24, n = 26; *P*<0.001). On the other hand, the additional *Alx4^Lst^* mutation induced the opposite effect (*Gli3^Xt/+^; Shh^+/−^* versus *Gli3^Xt/+^; Alx4^Lst/+^; Shh^+/−^*: 0.90±0.24, n = 26 versus 2.03±0.74, n = 10; *P*<0.001). From these results, we suggest that both *Gli3* and *Alx4* genes may negatively regulate Hh signaling.

### Compound allelic series of *Alx4* and *Gli3* mutants display omphalocele and pelvic girdle abnormalities

**Figure 3:**
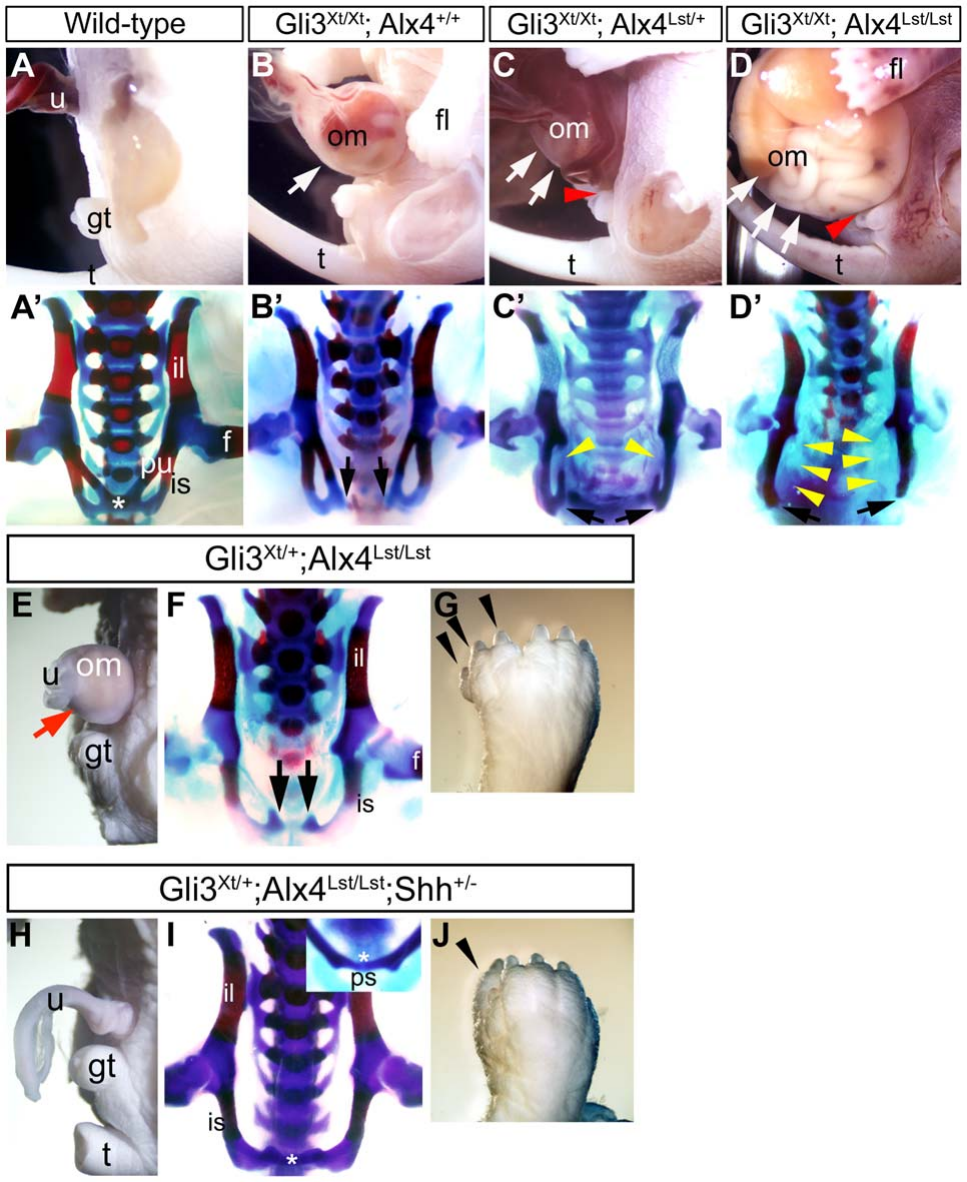
The omphalocele, pubic diastasis, loss of pubic bones and polydactyly in *Gli3^Xt^; Alx4^Lst^; Shh* combinatorial mutants. The lateral view of the embryonic ventral body wall (**A–D, E, H**) and frontal view of the pelvic girdle (**A'–D', F, I**). *Gli3^Xt/Xt^* embryos (**B**), *Gli3^Xt/Xt^; Alx4^Lst/+^* embryos (**C**) and *Gli3^Xt/Xt^; Alx4^Lst/Lst^* embryos (**D**) showed a graded extent of omphaloceles by the introduction of additional *Alx4^Lst^* alleles into the *Gli3^Xt/Xt^* background (**B–D**; white arrows). The dorsal parts of the genital tubercle were hypoplastic in *Gli3^Xt/Xt^; Alx4^Lst/+^* and *Gli3^Xt/Xt^; Alx4^Lst/Lst^* embryos (**C, D**; red arrowheads). The pubic symphysis of wild-type embryo was already formed at E18.5 (**A**'; asterisk). Pubic diastasis also became evident by the introduction of *Alx4^Lst^* mutation (**B'–D'**). *Gli3^Xt/Xt^; Alx4^Lst/+^* and *Gli3^Xt/Xt^; Alx4^Lst/Lst^* embryos showed partial loss of pubic bone components (**C', D'**; yellow arrowheads). Black arrows indicate the unclosed pelvis. *Gli3^Xt/+^; Alx4^Lst/Lst^* embryos showed omphalocele (**E**; red arrow), severe polydactyly (**G**), pubic diastasis and loss of pubic bones (**F**). Black arrows show the unclosed pelvis. *Gli3^Xt/+^; Alx4^Lst/Lst^; Shh^+/−^* embryos did not show omphalocele phenotypes (**H**). The pubic symphysis was formed but pubic bones were lost (**I**; asterisk). Polydactyly was still observed in *Gli3^Xt/+^; Alx4^Lst/Lst^; Shh^+/−^* embryos (**J**). Black arrowheads indicate extra digits. f: femur, fl: fore limb, gt: genital tubercle, il: iliac bone, is: ischial bone, om: omphalocele, ps: pubic symphysis, pu: pubic bone, t: tail, u: umbilical cord.

We generated graded levels of mutations for Hh signaling by introducing the *Alx4^Lst^* allele into a *Gli3^Xt/Xt^* background, and analyzed the resultant compound mutant embryos at E18.5 (Fig. 3A–D,A'–D'). The physiological umbilical hernia was recovered, and pubic symphysis was formed in wild-type embryos at E18.5 (Fig. 1D,I and Fig. 3A,A'). Decreasing wild-type *Alx4* alleles accelerated the degree of omphalocele in the *Gli3^Xt/Xt^* embryos (Fig. 3B–D). In *Gli3^Xt/Xt^; Alx4^Lst/+^* embryos and *Gli3^Xt/Xt^; Alx4^Lst/Lst^* embryos, the upper (dorsal) side of the genital tubercle was hypoplastic, in addition to the presence of an omphalocele (Fig. 3C,D). The development of the pelvic girdle also showed severe malformations in these mutants. The *Gli3^Xt/Xt^* embryos showed pubic diastasis (Fig. 3B'). The *Gli3^Xt/Xt^; Alx4^Lst/+^* embryos displayed pubic diastasis and partial loss of pubic bones (Fig. 3C'). The *Gli3^Xt/Xt^; Alx4^Lst/Lst^* embryos showed more severe truncation and separation of the pubic bones than the *Gli3^Xt/Xt^; Alx4^Lst/+^* embryos (Fig. 3D'). Thus, all of these mutants with omphalocele phenotypes displayed pubic diastasis.

### Phenotypic recovery of omphalocele and pubic diastasis, but not polydactyly and pubic bone hypoplasia, results from reducing the *Shh* allele

We further analyzed the effects of mutations in Hh signaling related genes. The *Gli3^Xt/+^; Alx4^Lst/Lst^* embryos also exhibited multiple deformities, including an omphalocele, polydactyly and the loss of pubic bones and their diastasis (Fig. 3E–G). Introducing a *Shh* mutation could restore some of these phenotypes in *Gli3^Xt/+^; Alx4^Lst/Lst^* embryos (Fig. 3H–J). The omphalocele observed in *Gli3^Xt/+^; Alx4^Lst/Lst^* embryos (Fig. 3E) was restored completely in *Gli3^Xt/+^; Alx4^Lst/Lst^; Shh^+/−^* embryos (Fig. 3H). On the other hand, polydactyly was partially rescued, but was still observed in these mice. While *Gli3^Xt/+^; Alx4^Lst/Lst^* embryos displayed polydactyly (Fig. 3G), the number of extra digits was reduced in the *Gli3^Xt/+^; Alx4^Lst/Lst^; Shh^+/−^* embryos (Fig. 3J). With regard to pelvic girdle development, parts of the pubic bones were still not observed but the midline symphysis of the pelvic girdle was formed in *Gli3^Xt/+^; Alx4^Lst/Lst^; Shh^+/−^* embryos (Fig. 3I; asterisk). Taken together, these results suggest the possible involvement of Hh signaling in omphalocele and pubic diastasis phenotypes.

### Ectopic Hh-signal activity is observed in *Gli3^Xt/Xt^* mutants

**Figure 4:**
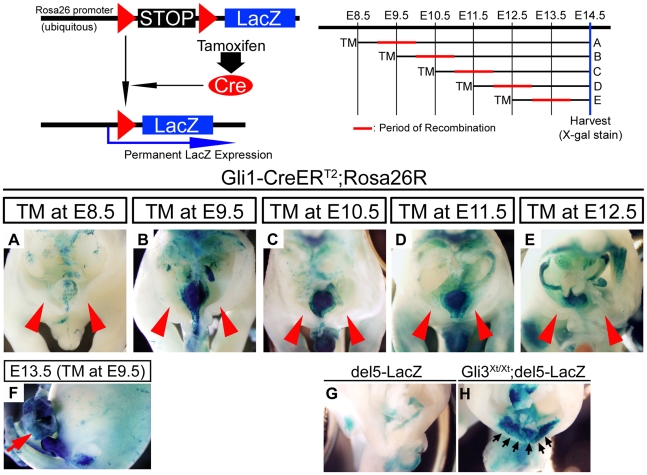
The analysis of Hh-responded cells in the ventral body wall region. A schematic diagram of Hh-responsive cell contribution assays. The *R26R* allele contains the LacZ gene and a floxed stop cassette under the *Rosa26* promoter. The *Gli1-CreER^T2^* allele contains an insertion of TM-inducible Cre recombinase into the *Gli1* gene locus. By crossing the *Gli1-CreER^T2^; Rosa26R* and ICR mice, gene recombination in Hh-responded cells could be achieved specifically under the control of TM. The embryos were stained with X-gal and dissected horizontally at the umbilical cord level. The stages of TM administration and estimated recombination periods in **A–E** are depicted. Under these TM treatment conditions, few LacZ-positive cells were observed in the ventral body wall (**A–E**; red arrowheads). The lateral view of the embryo also showed few Hh-responded cells (**F**). Red arrow indicates the LacZ-positive population in visceral organs. The *del5-LacZ* transgenic mice, the Hh signal indicator strain, displayed relatively high ectopic Hh signal activity in the *Gli3^Xt/Xt^* background compared with the control at E12.5 (**G**, **H**; black arrows).

In order to analyze the contribution of Hh-responded cells, we utilized the *Gli1-CreER^T2^; R26R* system. In *Gli1-CreER^T2^* mice, a TM-inducible form of Cre recombinase (*CreER^T2^*) was knocked into the *Gli1* locus (*Gli1-CreER^T2^*), which is one of the direct target genes of Hh signaling [60], [68], [69], [77]. The *Gli1-CreER^T2^* hemizygotes correspond to *Gli1^+/−^* mutants, and displayed normal morphology in the ventral body wall (data not shown). By crossing *Gli1-CreER^T2^/+; R26R/R26R* males and ICR females, we could obtain *Gli1-CreER^T2^/+; R26R/+* embryos. We treated pregnant ICR females once with 2 mg/40 g bw of TM at 8.5, 9.5, 10.5, 11.5 or 12.5 days post coitum, and embryos were collected at E14.5 (Fig. 4A–E) or at E13.5 (Fig. 4F). The recombination period in this system was estimated to occur within 6–12 hours and to continue for up to 36 hours after TM administration [60], [63]. Our protocols were expected to detect Hh-responded cells during an embryonic period approximately from E8.75 to E14.0. Under these TM treatment conditions, we could not detect a significant LacZ-positive population in the ventral body wall region (Fig. 4A–F).

We also employed a reporter mouse strain (*del5-LacZ*) to locate active Hh signaling *in vivo*. The *del5-LacZ* model employs Gli-responsive binding sites identified in the upstream sequence of the *Foxa2* gene [65], [66]. In the ventral body wall region, we could not observe Hh signal activities in the *del5-LacZ* strain at E12.5 (Fig. 4G). This result was consistent with Hh-responded cell contribution analysis. In contrast, we observed ectopic Hh activity by *del5-LacZ* staining with the *Gli3^Xt/Xt^* mutation at E12.5 (Fig. 4H). These results imply that Hh signaling may not play essential roles in normal development of the embryonic ventral body wall, but may be implicated in omphalocele pathogenesis.

### Augmented Hedgehog signaling results in omphalocele phenotypes

**Figure 5:**
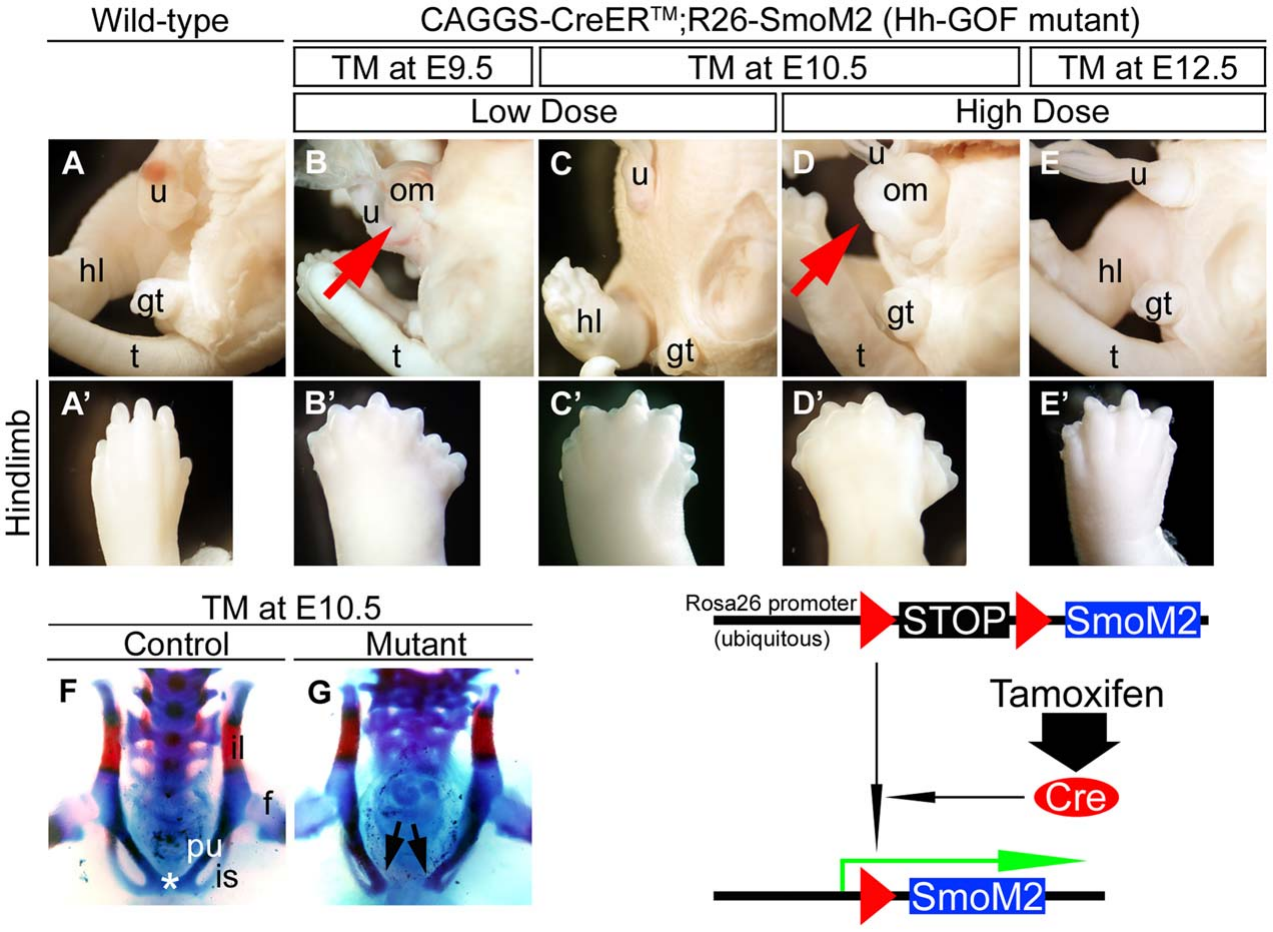
The conditional activation of Hh signaling by the protocols inducing omphalocele and pubic diastasis phenotypes. The *R26-SmoM2* allele contains the constitutively activated form of *Smoothened* and a floxed stop cassette under the control of the *Rosa26* promoter. By crossing *R26-SmoM2* mice and the TM-inducible form of Cre-driver mice, administration of TM to the pregnant mice induced embryonic stage-specific gene recombination, allowing continuous activation of Hh signaling. The lateral view of the body trunk and the left hind limb of a wild-type embryo treated with a high dose of TM at E10.5 (**A**, **A'**). Mutant embryos treated with a low dose of TM at E9.5 (**B**, **B'**), a low dose of TM at E10.5 (**C**, **C'**), a high dose of TM at E10.5 (**D**, **D'**) and a high dose of TM at E12.5 (**E**, **E**'). Embryos were collected at E17.5 (**A–G** and **A'–E'**). Mutant embryos treated with the low dose of TM at E9.5 and the high dose of TM at E10.5 showed omphalocele phenotypes (**B,** **D**; red arrows). Under such conditions, mutants displayed polydactyly phenotypes (**B'–E'**). Control embryos at E17.5 developed a pubic symphysis (**F**; asterisk). Mutant embryos treated with a high dose of TM at E10.5 showed a pubic diastasis phenotype (**G**). Black arrows indicate the unclosed pelvis. f: femur, gt: genital tubercle, hl: hind limb, il: iliac bone, is: ischial bone, om: omphalocele, pu: pubic bone, t: tail, u: umbilical cord.

To assess the effects of ectopically-induced Hh signaling, we analyzed gain-of-function mutants of Hh signaling (hereafter designated as Hh-GOF) by utilizing the TM-inducible gene recombination system. Ectopic induction of Hh signaling was achieved by utilizing *R26-SmoM2* and *CAGGS-CreER™* mice. The *CAGGS-CreER™* mice display Cre activity throughout the body upon TM treatment [63]. The *R26-SmoM2* allele possesses the constitutively activated form of *Smoothened* (*SmoM2*) and a floxed stop cassette under the ubiquitous *Rosa26* promoter [64], [73]. By crossing *R26-SmoM2* mice and the TM-inducible form of Cre-driver mice, activation of Hh signaling was achieved. We analyzed mutant embryos that were treated once with various doses of TM (1 mg, 2 mg or 4 mg/40 g bw) at various time points on E9.5, E10.5, E11.5, E12.5 or E13.5, respectively. No noticeable toxic effects were observed for any of these TM treatment protocols [65], [70], [71]. Upon administration of TM at E9.5, E10.5 or E11.5, mutant embryos displayed omphalocele and polydactyly phenotypes (Fig. 5B,B',D,D'; data not shown). The phenotypic differences induced by the different doses of TM were present following administration at E10.5 (Fig. 5C,C',D,D'). Omphaloceles were prominently observed in embryos from dams treated with the higher dose of TM (2 mg/40 g bw) but not with the lower dose (1 mg/40 g bw) (Fig. 5C,D). In contrast to the mutants treated with TM at E10.5, Hh-GOF mutants did not display an omphalocele even with the higher dose of TM treatment (4 mg/40 g bw) at E12.5 (Fig. 5E). On the other hand, the mutants exhibited an omphalocele induced by the lower dose of TM treatment (1 mg/40 g bw) at E9.5 (Fig. 5B). With regard to the phenotypes for digits and the pelvic girdle, the mutants with omphaloceles also showed severe polydactyly (Fig. 5B',D') compared with the non-omphalocele mutants in their hind limbs (Fig. 5C',E'). The pubic symphysis was formed in control embryos at E17.5 (Fig. 5F). The Hh-GOF mutants showed pubic diastasis when 2 mg/40 g bw of TM was administered at E10.5 (Fig. 5G). These results may indicate that the pathogenesis of omphalocele is induced by augmented Hh signaling in a time- and dose-dependent manner.

### Abnormal body wall muscle formation and excessive cell death would be associated with omphalocele formation in the Hh-GOF mutants

**Figure 6:**
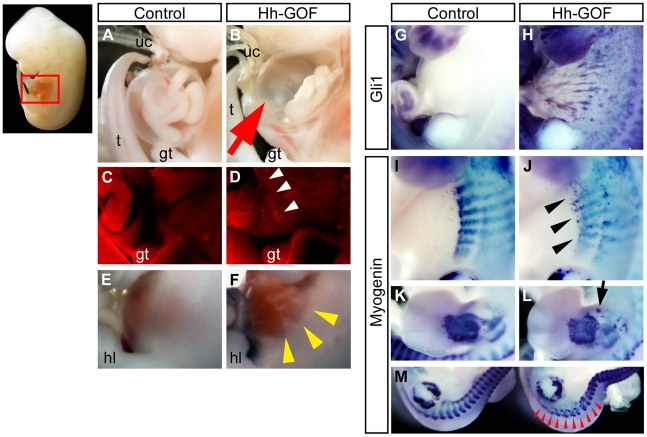
Formation of embryonic abdominal muscles and herniation of the visceral organs are affected by Hh signal activation. The *CAGGS-CreER™; R26-SmoM2* (Hh-GOF) mutant embryos showed a moderate degree of herniation into the sac (peritoneal membrane) of physiological umbilical hernia compared with control embryos (**A**, **B**; red arrow). Hh-GOF mutants also exhibited more prominent cell death compared with controls as determined by acridine orange staining (**C**, **D**; white arrowheads). In addition, the lateral embryonic trunk was malformed in mutants (**E**, **F**; yellow arrowheads). Expression analysis of *Gli1* confirmed the ectopic induction of Hh signaling in the ventral body wall (**G**, **H**). *Myogenin* expression was weaker (**J**; black arrowheads) and ectopically located (**L**; black arrow) in Hh-GOF mutants (**I–M**). Red arrowheads indicate affected muscle precursors after Hh activation.

We further analyzed the Hh-GOF mutants in mid-gestation. To confirm the induction of ectopic Hh signaling, we performed gene expression analyses as one of the readouts of Hh signaling: *Gli1* mRNA in Hh-GOF mutant embryos. The expression of *Gli1* was observed ectopically throughout the body, including the lateral body wall in Hh-GOF mutants (Fig. 6H). We hypothesized that two potential causative factors might underlie the etiology of omphalocele formation. One could be an abnormality in the endodermal organs, such as an excess bulging out of visceral organs when the physiological umbilical hernia is observed. Another possibility could be defects in the mesodermal or ectodermal organs, such as a failure of the ventral body wall muscle formation. We expected that either or both of these factors could cause omphalocele formation. To assess these possibilities, we analyzed *CAGGS-CreER™; R26-SmoM2* embryos using different TM administration protocols (TM treatment with 2 mg/40 g bw at E10.5 and harvested at E14.5, or TM treatment with 1 mg/40 g bw at E9.5 and harvested at E12.5 and E13.5). These TM administration protocols were sufficient to induce an omphalocele in later embryonic stages, and all of these mutants exhibited similar phenotypes (Fig. 5B,D). Interestingly, the volume of the herniating viscera in the peritoneal sac appeared smaller in Hh-GOF mutants than in control embryos (Fig. 6A,B; red arrow). On the other hand, excessive amount of cell death was detected by acridine orange staining in Hh-GOF mutants during the ventral body wall formation (Fig. 6D). With regard to the muscle differentiation, gene expression analyses of a muscle marker, *Myogenin*, suggested that the populations of muscle precursors in both the lateral body wall and limbs were decreased and distributed abnormally in Hh-GOF mutants (Fig. 6I–L). The lateral body wall of the mutants seemed to be disorganized (Fig. 6F; yellow arrowheads). Moreover, both epaxial and hypaxial muscle precursors seemed to be affected in Hh-GOF mutants (Fig. 6M; red arrowheads). These results might suggest that the pathogenesis of omphalocele in Hh-GOF mutants could be due to the failure of body wall formation and an abnormally enlarged umbilical ring associated with excessive cell death.

## Discussion

Recent advances in developmental biology and human embryology provide a profound understanding of the organogenesis and the pathogenesis of congenital diseases [78]. Embryonic organogenesis is potentially influenced by genetic programs, maternal-embryonic interactions and embryonic physiological conditions [79], [80]. The development of the ventral body wall displays dynamic processes, such as that observed during the formation and recovery of the physiological umbilical hernia. These processes proceed with the proper formation of adjacent structures, including body wall muscles and the pelvic girdle. Although some previous studies of genetically-modified animals have reported the processes of ventral body wall formation, our understanding of the ventral body wall formation and its related dysmorphogenesis remains incomplete. We herein reported that Hedgehog signaling is one of the causative factors for omphalocele formation, as demonstrated by utilizing a series of combinatorial mutants for Hh signaling genes and conditional gain-of-function mutants of the Hh signaling pathway. The analyses of *Shh, Gli3* and *Alx4* compound mutant embryos revealed that the introduction of additional *Alx4^Lst^* mutations into the *Gli3^Xt/Xt^* background resulted in the corresponding omphalocele and pubic diastasis. Moreover, the reduction of a single *Shh* allele restored omphalocele and pubic symphysis formation in the *Gli3^Xt/+^; Alx4^Lst/Lst^* embryos. The *CAGGS-CreER™; R26-SmoM2* (Hh-GOF) conditional mutant analyses revealed the Hh signal-dependent omphalocele formation and pubic diastasis. This would therefore be the first demonstration of the involvement of Hh signaling in ventral body wall malformation and the genetic rescue of omphalocele formation.

### The possible factors causing omphalocele formation

In spite of recent advances in embryology and pathology, the etiology of omphalocele formation is still controversial. It has been suggested that a failure of the gut to return to the abdominal cavity after physiological herniation at appropriate developmental stages results in an omphalocele [6], [10], [81]. According to this hypothesis, the lateral body wall closure has not been considered to be related to omphalocele formation, because the loops of the bowel are in the cord and are covered by amniotic membranes [9]. Another possible cause of omphalocele could be a midline defect at the amnio-ectodermal transition, the transition zone between the ectoderm and mesoderm, which would result in an enlarged umbilical ring [2], [7], [11], [12]. During normal development, a mature body wall covers the ventral surface surrounding the ring and the cord. With omphalocele, the mature body wall shows incomplete closure and it is localized to the periphery of the enlarged umbilical ring [82].

Our current studies suggested that few Hh-responded cells could contribute to the normal ventral body wall formation (Fig. 4A–F). In contrast to such results, we observed the ectopic Hh signaling in *Gli3^Xt/Xt^* mutants by utilizing the *del5-LacZ* Hh reporter mouse strain (Fig. 4H). In the *Alx4^Lst/Lst^* mutants, *Shh* expression was augmented in the cloacal epithelium, and ectopic Hh signal activity was observed in the ventral body wall region by *del5-LacZ* staining (data not shown). Moreover, gain-of-function mutants of Hh signaling displayed defects in body wall formation (Fig. 6F,J). These results suggested that omphalocele might be caused by ectopically-induced Hh signaling.

In this manuscript, we utilized the system for tamoxifen inducible ubiquitous activation of Hh signaling by *CAGGS-CreER™; R26-SmoM2*. However, the identification of such Hh-responded tissues remained unclear even when utilizing this conditional activation system. Hence, we further analyzed mutants that display specific activation of Hh signaling in the endodermal organs. To achieve endodermal activation of Hh signaling, *Gli1-CreER^T2^; R26-SmoM2* mice and *Shh-CreER^T2^; R26-SmoM2* mice were employed. The *Gli1-CreER^T2^* mice and *Shh-CreER^T2^* mice possess a tamoxifen-inducible form of Cre recombinase in the *Gli1* and *Shh* gene loci, respectively [60], [61]. *Shh* is specifically expressed in the endodermal epithelia of many visceral organs, and *Gli1* is expressed mainly in the mesenchyme of visceral organs (Fig. S1A–C) [83]. Regardless of the identity of Cre-driver lines, none of the mutants displayed omphalocele phenotypes (Fig. S2; data not shown). These results may suggest that Hh signal activation in endodermal organs may not be sufficient to induce such phenotypes under the current experimental conditions. In addition, the *Gli1* gene is also considered to be one of the direct target genes of Hh signaling [60], [84], [85]. Hence, the allelic combination of *Gli1-CreER^T2^; R26-SmoM2* could result in Hh signal activation in Hh-responded tissues in such mutant embryos. Based on comparison of the phenotypes of *Gli1-CreER^T2^; R26-SmoM2* and *CAGGS-CreER™; R26-SmoM2* embryos, we would also suggest that the pathogenesis of the omphalocele was due to ectopically-induced Hh signaling (Fig. 5D and Fig. S2A').

With regard to the involvement of various mesodermal and ectodermal tissues in the pathogenesis of omphalocele formation, our previous study showed that there were abdominal wall defects with disorganized muscle layers and connective tissues in *Msx1/2* double mutant mice [28]. Likewise, abnormal *Alk3*-mediated BMP signaling in mesodermal tissues caused prenatal omphalocele-like defects [26]. Moreover, the phenotypes of *Pitx2* knockout mice and *PITX2* mutation in humans indicate a correlation with the omphalocele formation [33], [86]. The *Pitx2* gene, which is expressed in abdominal muscles such as the rectus abdominis and oblique abdominis, has pivotal roles in muscle anlagen formation and maintenance [23], [87]–[89]. Altogether, these reports suggest essential roles of mesodermal or ectodermal tissues in omphalocele formation.

Another question arises regarding the onset of omphalocele formation. We showed the presence of a critical time-window for inducing omphalocele phenotypes by analyzing temporally-regulated conditional mutants of *CAGGS-CreER™; R26-SmoM2* mice (Fig. 5B,D,E). Hh signal induction before the stage of physiological umbilical herniation (E9.5 and E10.5) resulted in omphalocele formation (Fig. 5B,D). In addition, activation of Hh signaling by TM injection at E12.5 did not lead to omphalocele formation (Fig. 5E). These results may imply that the onset of omphalocele formation thus begins before physiological umbilical herniation.

### The formation of pubic symphysis may be related to body wall dysmorphogenesis

The pathogenic sequences of human patients with dysgenesis of the bladder and external genitalia have been reported. Such phenotypes often display exstrophy of the bladder and hypoplasia of the upper (dorsal) side of the penis [90], [91]. Another characteristic symptom of bladder exstrophy is pubic diastasis (pelvic girdle separation) [15], [92]. These complex congenital defects prompted the idea of coordinated urogenital organ development [18], [65]. Despite the significant correlations of clinical conditions, the influence of pelvic girdle formation on such developmental coordination has previously been little analyzed.

Our current study revealed the concordant recovery of the physiological umbilical hernia and the closure of pelvic girdle at late embryonic stages (from E14.5 to E16.5) (Fig. 1B,C,G,H). According to textbook anatomy, the rectus abdominis arises from the front of the pubic symphysis and from the pubic crest [93], [94]. Myocytes of the abdominal musculature are of somitic origin and the connective tissue elements within the abdominal wall, including tendons, derive from the somatopleure [95]. Moreover, the entire pelvic elements also originate from the somatopleure [76], [96]. We suggested that both body wall muscle primordia and pelvic girdle primordia arise from the lateral side of the embryonic trunk and develop toward the midline of the body (Fig. 1E–I and Fig. S3A–C). These observations imply that an unclosed pelvic girdle may be related to the phenotype with unclosed body wall muscles. In fact, *Gli3^Xt/Xt^* and *Alx4^Lst/Lst^* embryos displayed the absence of the midline muscular structures (Fig. S4C,D).

Hh signaling can affect the formation of midline structures in many developmental contexts of mammalian embryos, such as craniofacial formation. For instance, decreased Hh signal activity causes holoprosencephaly and hypotelorism, in contrast to augmented Hh signaling, which causes hypertelorism and frontonasal dysplasia [97]. Our studies may also be supported by a broad spectrum of observations covering several processes of organogenesis. In the current study, the mutants with omphalocele phenotypes tended to show pubic diastasis. We revealed a possible association between the degree of omphalocele phenotype and pubic diastasis phenotype (Fig. 3B–D,B'–D'). In addition, mutants genetically rescued from omphalocele (*Gli3^Xt/+^; Alx4^Lst/Lst^; Shh^+/−^*) did form the pubic symphysis and normal midline muscle structures (Fig. 3I; data not shown). These results may indicate a role for Hh signaling in the formation of such midline structures, and a developmental correlation between the ventral body wall and the pelvic girdle.

### The extent of dysmorphogenesis in the body wall, pelvic girdle and digit formation by ectopically-induced Hedgehog signaling

While abnormal Hh signaling has been implicated as one of the major causes of polydactyly in mice, its involvement in the omphalocele formation has so far not been examined. In the current study, we showed that reduction of a single *Shh* allele could restore the omphalocele but could not restore the polydactyly of *Gli3^Xt/+^; Alx4^Lst/Lst^* embryos (Fig. 3H,J). In addition, the Hh-GOF mutants (*CAGGS-CreER™; R26-SmoM2*) without the omphalocele phenotype still exhibited polydactyly (Fig. 5C',E'). These results may suggest that the pathogenesis of the polydactyly phenotype seemed to be more sensitive to ectopically-induced Hh signaling than that of the omphalocele phenotype. In support of this notion, the frequency of polydactyly is higher than omphalocele, and has been reported to occur in approximately 1 in 600 human births [98]. On the other hand, the frequency of omphalocele is relatively low, namely approximately 1 in 4,000 human births [6].

In the current study, we suggested that ectopically-induced Hh signaling might be one of the causes of a combination of polydactyly, omphalocele and pubic diastasis phenotypes. These results may offer a clue that can help elucidate the mechanisms underlying the formation of omphalocele and its associated syndromic malformations.

## Supporting Information

Figure S1 **Cre recombinase activities of *Shh-CreER^T2^* and *Gli1-CreER^T2^in the developing gut.*** The *Shh-CreER^T2^* activity was not observed in the ventral body wall at E14.5 upon tamoxifen treatment (4 mg/40 g maternal body weight) at E9.5 (**A**). Red arrow indicates the expression in the developing gut. The expression of Cre recombinase was detected in the endodermal epithelia of the midgut and posterior part of limb buds (**B**). The activity of *Gli1-CreER^T2^* was also observed in the embryonic gut, including a part of the mesentery (**C**). The *Gli1-CreER^T2^; R26R* embryo was treated with 4 mg/40 g bw of tamoxifen at E10.5 and harvested at E13.5.
(TIF)

Figure S2 **Augmentation of Hh signaling by utilizing *Gli1-CreER^T2^* and *Shh-CreER^T2^*driver mouse lines.** Both *Gli1-CreER^T2^; R26-SmoM2* and *Shh-CreER^T2^; R26-SmoM2* embryos did not display omphalocele phenotypes following administration of 4 mg/40 g bw of tamoxifen at E10.5 (**A**, **A**', **B**, **B**'). gt: genital tubercle, hl: hind limb, t: tail, uc: umbilical cord.
(TIF)

Figure S3 **The expression of *Myogenin* in wild-type embryos at E10.5, E11.5 and E12.5.** The ratio between primordia of hypaxial musculature (a, a' and a″) and epaxial musculature (b, b' and b″) was gradually increased during these stages, as hypaxial musculature (body wall muscle precursors) developed toward the midline (**A-C**).
(TIF)

Figure S4 **Absence of midline structures in *Alx4^Lst/Lst^ and *Gli3^Xt/Xt^** embryos.** Sagittal sections of a control embryo at E18.5 displayed prominent pubic symphysis (**A**; asterisk) and abdominal muscle structures (**B**). Muscles were stained with Anti-Skeletal Myosin antibody (FAST) (Sigma). Neither *Alx4^Lst/Lst^* (**C**) nor *Gli3^Xt/Xt^* (**D**) mutant embryos developed pubic symphysis or abdominal muscles, as shown by sagittal sections. b: bladder, gt: genital tubercle, om: omphalocele, r: rectum, u: urethra.
(TIF)

## References

[1] Kaufman MH (1992). The
atlas of mouse development..

[2] Brewer S, Williams T (2004). Finally,
a sense of closure? Animal models of human ventral body wall defects.. Bioessays.

[3] Eggenschwiler J, Ludwig T, Fisher P, Leighton P, Tilghman S (1997). Mouse
mutant embryos overexpressing IGF-II exhibit phenotypic features of the Beckwith-Wiedemann
and Simpson-Golabi-Behmel syndromes.. Genes
Dev.

[4] Achiron R, Soriano D, Lipitz S, Mashiach S, Goldman B (1995). Fetal
midgut herniation into the umbilical cord: improved definition of ventral
abdominal anomaly with the use of transvaginal sonography.. Ultrasound Obstet Gynecol.

[5] Mann S, Blinman T, Douglas Wilson R (2008). Prenatal
and postnatal management of omphalocele.. Prenat
Diagn.

[6] Sadler T (2006). Langman's
medical embryology..

[7] Glasser JG (2009). Omphalocele
and Gastroschisis..

[8] Weber T, Au-Fliegner M, Downard C, Fishman S (2002). Abdominal
wall defects.. Curr Opin Pediatr.

[9] Sadler T (2010). The
embryologic origin of ventral body wall defects.. Semin Pediatr Surg.

[10] Mesaeli N, Nakamura K, Zvaritch E, Dickie P, Dziak E (1999). Calreticulin
is essential for cardiac development.. J
Cell Biol.

[11] Taeusch HW, Ballard RA, Gleason CA, Avery ME (2005). Avery's
diseases of the newborn..

[12] Hirano M, Kiyonari H, Inoue A, Furushima K, Murata T (2006). A
new serine/threonine protein kinase, Omphk1, essential to ventral body wall
formation.. Dev Dyn.

[13] Yazbeck S, Ndoye M, Khan AH (1986). Omphalocele:
a 25-year experience.. J Pediatr Surg.

[14] Aspelund G, Langer J (2006). Abdominal
wall defects.. Current Paediatrics.

[15] Perovic SV (1999). Atlas
of Congenital Anomalies of the External Genitalia; Perovic, V. S, editors..

[16] Langer J (2003). Abdominal
wall defects.. World J Surg.

[17] Ludwig M, Ching B, Reutter H, Boyadjiev S (2009). Bladder
exstrophy-epispadias complex.. Birth Defects
Res A Clin Mol Teratol.

[18] Suzuki K, Economides A, Yanagita M, Graf D, Yamada G (2009). New
horizons at the caudal embryos: coordinated urogenital/reproductive organ
formation by growth factor signaling.. Curr
Opin Genet Dev.

[19] Carey J (2001). Exstrophy
of the cloaca and the OEIS complex: one and the same.. Am J Med Genet.

[20] Bohring A (2002). OEIS
complex, VATER, and the ongoing difficulties in terminology and delineation.. Am J Med Genet.

[21] Stepan H, Horn L, Bennek J, Faber R (1999). Congenital
hernia of the abdominal wall: a differential diagnosis of fetal abdominal
wall defects.. Ultrasound Obstet Gynecol.

[22] Lin C, Kioussi C, O'Connell S, Briata P, Szeto D (1999). Pitx2
regulates lung asymmetry, cardiac positioning and pituitary and tooth morphogenesis.. Nature.

[23] Kitamura K, Miura H, Miyagawa-Tomita S, Yanazawa M, Katoh-Fukui Y (1999). Mouse
Pitx2 deficiency leads to anomalies of the ventral body wall, heart, extra-
and periocular mesoderm and right pulmonary isomerism.. Development.

[24] Nottoli T, Hagopian-Donaldson S, Zhang J, Perkins A, Williams T (1998). AP-2-null
cells disrupt morphogenesis of the eye, face, and limbs in chimeric mice.. Proc Natl Acad Sci U S A.

[25] Dunker N, Krieglstein K (2002). Tgfbeta2 −/−
Tgfbeta3 −/− double knockout mice display severe midline fusion
defects and early embryonic lethality.. Anat
Embryol (Berl).

[26] Sun J, Liu YH, Chen H, Nguyen MP, Mishina Y (2007). Deficient
Alk3-mediated BMP signaling causes prenatal omphalocele-like defect.. Biochem Biophys Res Commun.

[27] Goldman D, Hackenmiller R, Nakayama T, Sopory S, Wong C (2006). Mutation
of an upstream cleavage site in the BMP4 prodomain leads to tissue-specific
loss of activity.. Development.

[28] Ogi H, Suzuki K, Ogino Y, Kamimura M, Miyado M (2005). Ventral
abdominal wall dysmorphogenesis of Msx1/Msx2 double-mutant mice.. Anat Rec A Discov Mol Cell Evol Biol.

[29] Brewer S, Williams T (2004). Loss
of AP-2alpha impacts multiple aspects of ventral body wall development and
closure.. Dev Biol.

[30] Schorle H, Meier P, Buchert M, Jaenisch R, Mitchell P (1996). Transcription
factor AP-2 essential for cranial closure and craniofacial development.. Nature.

[31] Zhang J, Hagopian-Donaldson S, Serbedzija G, Elsemore J, Plehn-Dujowich D (1996). Neural
tube, skeletal and body wall defects in mice lacking transcription factor
AP-2.. Nature.

[32] Lu M, Pressman C, Dyer R, Johnson R, Martin J (1999). Function
of Rieger syndrome gene in left-right asymmetry and craniofacial development.. Nature.

[33] Gage P, Suh H, Camper S (1999). Dosage
requirement of Pitx2 for development of multiple organs.. Development.

[34] Varjosalo M, Taipale J (2008). Hedgehog:
functions and mechanisms.. Genes Dev.

[35] Riddle R, Johnson R, Laufer E, Tabin C (1993). Sonic
hedgehog mediates the polarizing activity of the ZPA.. Cell.

[36] Jiang J, Hui C (2008). Hedgehog
signaling in development and cancer.. Dev
Cell.

[37] McGlinn E, Tabin C (2006). Mechanistic
insight into how Shh patterns the vertebrate limb.. Curr Opin Genet Dev.

[38] Zhu J, Nakamura E, Nguyen M, Bao X, Akiyama H (2008). Uncoupling
Sonic hedgehog control of pattern and expansion of the developing limb bud.. Dev Cell.

[39] Zeller R, López-Ríos J, Zuniga A (2009). Vertebrate
limb bud development: moving towards integrative analysis of organogenesis.. Nat Rev Genet.

[40] te Welscher P, Zuniga A, Kuijper S, Drenth T, Goedemans H (2002). Progression
of vertebrate limb development through SHH-mediated counteraction of GLI3.. Science.

[41] Litingtung Y, Dahn R, Li Y, Fallon J, Chiang C (2002). Shh
and Gli3 are dispensable for limb skeleton formation but regulate digit number
and identity.. Nature.

[42] Milenkovic L, Goodrich L, Higgins K, Scott M (1999). Mouse
patched1 controls body size determination and limb patterning.. Development.

[43] Butterfield N, Metzis V, McGlinn E, Bruce S, Wainwright B (2009). Patched
1 is a crucial determinant of asymmetry and digit number in the vertebrate
limb.. Development.

[44] Mao J, Barrow J, McMahon J, Vaughan J, McMahon A (2005). An
ES cell system for rapid, spatial and temporal analysis of gene function in
vitro and in vivo.. Nucleic Acids Res.

[45] Pan Y, Wang C, Wang B (2009). Phosphorylation
of Gli2 by protein kinase A is required for Gli2 processing and degradation
and the Sonic Hedgehog-regulated mouse development.. Dev Biol.

[46] Ovchinnikov DA, Selever J, Wang Y, Chen YT, Mishina Y (2006). BMP
receptor type IA in limb bud mesenchyme regulates distal outgrowth and patterning.. Dev Biol.

[47] Lallemand Y, Nicola MA, Ramos C, Bach A, Cloment CS (2005). Analysis
of Msx1; Msx2 double mutants reveals multiple roles for Msx genes in limb
development.. Development.

[48] Bassett E, Williams T, Zacharias A, Gage P, Fuhrmann S (2010). AP-2alpha
knockout mice exhibit optic cup patterning defects and failure of optic stalk
morphogenesis.. Hum Mol Genet.

[49] Hill P, Gotz K, Ruther U (2009). A
SHH-independent regulation of Gli3 is a significant determinant of anteroposterior
patterning of the limb bud.. Dev Biol.

[50] Hui CC, Joyner AL (1993). A
mouse model of greig cephalopolysyndactyly syndrome: the extra-toesJ mutation
contains an intragenic deletion of the Gli3 gene.. Nat Genet.

[51] Kim P, Mo R, Hui Cc C (2001). Murine
models of VACTERL syndrome: Role of sonic hedgehog signaling pathway.. J Pediatr Surg.

[52] Panman L, Drenth T, Tewelscher P, Zuniga A, Zeller R (2005). Genetic
interaction of Gli3 and Alx4 during limb development.. Int J Dev Biol.

[53] Qu S, Niswender KD, Ji Q, van der Meer R, Keeney D (1997). Polydactyly
and ectopic ZPA formation in Alx-4 mutant mice.. Development.

[54] Qu S, Tucker SC, Ehrlich JS, Levorse JM, Flaherty LA (1998). Mutations
in mouse Aristaless-like4 cause Strong's luxoid polydactyly.. Development.

[55] Takahashi M, Tamura K, Buscher D, Masuya H, Yonei-Tamura S (1998). The
role of Alx-4 in the establishment of anteroposterior polarity during vertebrate
limb development.. Development.

[56] Kuijper S, Feitsma H, Sheth R, Korving J, Reijnen M (2005). Function
and regulation of Alx4 in limb development: complex genetic interactions with
Gli3 and Shh.. Dev Biol.

[57] Masuya H, Sagai T, Moriwaki K, Shiroishi T (1997). Multigenic
control of the localization of the zone of polarizing activity in limb morphogenesis
in the mouse.. Dev Biol.

[58] Kuijper S, Beverdam A, Kroon C, Brouwer A, Candille S (2005). Genetics
of shoulder girdle formation: roles of Tbx15 and aristaless-like genes.. Development.

[59] Chiang C, Litingtung Y, Lee E, Young KE, Corden JL (1996). Cyclopia
and defective axial patterning in mice lacking Sonic hedgehog gene function.. Nature.

[60] Ahn S, Joyner A (2004). Dynamic
changes in the response of cells to positive hedgehog signaling during mouse
limb patterning.. Cell.

[61] Harfe B, Scherz P, Nissim S, Tian H, McMahon A (2004). Evidence
for an expansion-based temporal Shh gradient in specifying vertebrate digit
identities.. Cell.

[62] Soriano P (1999). Generalized
lacZ expression with the ROSA26 Cre reporter strain.. Nat Genet.

[63] Hayashi S, McMahon AP (2002). Efficient
recombination in diverse tissues by a tamoxifen-inducible form of Cre: a tool
for temporally regulated gene activation/inactivation in the mouse.. Dev Biol.

[64] Mao J, Ligon KL, Rakhlin EY, Thayer SP, Bronson RT (2006). A
novel somatic mouse model to survey tumorigenic potential applied to the Hedgehog
pathway.. Cancer Res.

[65] Haraguchi R, Motoyama J, Sasaki H, Satoh Y, Miyagawa S (2007). Molecular
analysis of coordinated bladder and urogenital organ formation by Hedgehog
signaling.. Development.

[66] Sasaki H, Hui C, Nakafuku M, Kondoh H (1997). A
binding site for Gli proteins is essential for HNF-3beta floor plate enhancer
activity in transgenics and can respond to Shh in vitro.. Development.

[67] Danielian PS, Muccino D, Rowitch DH, Michael SK, McMahon AP (1998). Modification
of gene activity in mouse embryos in utero by a tamoxifen-inducible form of
Cre recombinase.. Curr Biol.

[68] Feil R, Wagner J, Metzger D, Chambon P (1997). Regulation
of Cre recombinase activity by mutated estrogen receptor ligand-binding domains.. Biochem Biophys Res Commun.

[69] Feil R, Brocard J, Mascrez B, LeMeur M, Metzger D (1996). Ligand-activated
site-specific recombination in mice.. Proc
Natl Acad Sci U S A.

[70] Miyagawa S, Moon A, Haraguchi R, Inoue C, Harada M (2009). Dosage-dependent
hedgehog signals integrated with Wnt/beta-catenin signaling regulate external
genitalia formation as an appendicular program.. Development.

[71] Miyagawa S, Satoh Y, Haraguchi R, Suzuki K, Iguchi T (2009). Genetic
interactions of the androgen and Wnt/beta-catenin pathways for the masculinization
of external genitalia.. Mol Endocrinol.

[72] Ahn S, Joyner A (2005). In
vivo analysis of quiescent adult neural stem cells responding to Sonic hedgehog.. Nature.

[73] Jeong J, Mao J, Tenzen T, Kottmann AH, McMahon AP (2004). Hedgehog
signaling in the neural crest cells regulates the patterning and growth of
facial primordia.. Genes Dev.

[74] Haraguchi R, Mo R, Hui C, Motoyama J, Makino S (2001). Unique
functions of Sonic hedgehog signaling during external genitalia development.. Development.

[75] Suzuki K, Yamaguchi Y, Villacorte M, Mihara K, Akiyama M (2009). Embryonic
hair follicle fate change by augmented {beta}-catenin through Shh and Bmp
signaling.. Development.

[76] Pomikal C, Streicher J (2010). 4D-analysis
of early pelvic girdle development in the mouse (Mus musculus).. J Morphol.

[77] Indra A, Warot X, Brocard J, Bornert J, Xiao J (1999). Temporally-controlled
site-specific mutagenesis in the basal layer of the epidermis: comparison
of the recombinase activity of the tamoxifen-inducible Cre-ER(T) and Cre-ER(T2)
recombinases.. Nucleic Acids Res.

[78] Chan KK, Wong CK, Lui VC, Tam PK, Sham MH (2003). Analysis
of SOX10 mutations identified in Waardenburg-Hirschsprung patients: Differential
effects on target gene regulation.. J Cell
Biochem.

[79] Chan K, Chen Y, Yau T, Fu M, Lui V (2005). Hoxb3
vagal neural crest-specific enhancer element for controlling enteric nervous
system development.. Dev Dyn.

[80] Habib H, Hatta T, Rahman O, Yoshimura Y, Otani H (2007). Fetal
jaw movement affects development of articular disk in the temporomandibular
joint.. Congenit Anom (Kyoto).

[81] Feldkamp M, Carey J, Sadler T (2007). Development
of gastroschisis: review of hypotheses, a novel hypothesis, and implications
for research.. Am J Med Genet A.

[82] Williams T (2008). Animal
models of ventral body wall closure defects: a personal perspective on gastroschisis.. Am J Med Genet C Semin Med Genet.

[83] Kolterud A, Grosse A, Zacharias W, Walton K, Kretovich K (2009). Paracrine
Hedgehog signaling in stomach and intestine: new roles for hedgehog in gastrointestinal
patterning.. Gastroenterology.

[84] Dai P, Akimaru H, Tanaka Y, Maekawa T, Nakafuku M (1999). Sonic
Hedgehog-induced activation of the Gli1 promoter is mediated by GLI3.. J Biol Chem.

[85] Bai C, Auerbach W, Lee J, Stephen D, Joyner A (2002). Gli2,
but not Gli1, is required for initial Shh signaling and ectopic activation
of the Shh pathway.. Development.

[86] Katz L, Schultz R, Semina E, Torfs C, Krahn K (2004). Mutations
in PITX2 may contribute to cases of omphalocele and VATER-like syndromes.. Am J Med Genet A.

[87] Shih H, Gross M, Kioussi C (2007). Expression
pattern of the homeodomain transcription factor Pitx2 during muscle development.. Gene Expr Patterns.

[88] Kioussi C, Briata P, Baek S, Rose D, Hamblet N (2002). Identification
of a Wnt/Dvl/beta-Catenin —> Pitx2 pathway mediating cell-type-specific
proliferation during development.. Cell.

[89] Hilton T, Gross M, Kioussi C (2010). Pitx2-dependent
occupancy by histone deacetylases is associated with T-box gene regulation
in mammalian abdominal tissue.. J Biol
Chem.

[90] Mingin G, Nguyen H, Mathias R, Shepherd J, Glidden D (2002). Growth
and metabolic consequences of bladder augmentation in children with myelomeningocele
and bladder exstrophy.. Pediatrics.

[91] Ebert A, Reutter H, Ludwig M, Rösch W (2009). The
exstrophy-epispadias complex.. Orphanet
J Rare Dis.

[92] Sponseller P, Bisson L, Gearhart J, Jeffs R, Magid D (1995). The
anatomy of the pelvis in the exstrophy complex.. J Bone Joint Surg Am.

[93] Kawamura DM (1997). Abdomen
and superficial structures..

[94] Snell RS (1992). Clinical
anatomy for medical students..

[95] Christ B, Jacob M, Jacob H (1983). On
the origin and development of the ventrolateral abdominal muscles in the avian
embryo. An experimental and ultrastructural study.. Anat Embryol (Berl).

[96] Malashichev Y, Christ B, Pröls F (2008). Avian
pelvis originates from lateral plate mesoderm and its development requires
signals from both ectoderm and paraxial mesoderm.. Cell Tissue Res.

[97] Brugmann S, Allen N, James A, Mekonnen Z, Madan E (2010). A
primary cilia-dependent etiology for midline facial disorders.. Hum Mol Genet.

[98] Novick C, Grogan DP (2009). Polydactyly
of the Foot..

