# Supplementary figures and images for: Genetic Analysis of Hedgehog Signaling in Ventral Body Wall Development and the Onset of Omphalocele Formation
Source: PLoS One. 2011 Jan 20;6(1):e16260. doi: 10.1371/journal.pone.0016260 (PMC3024424; doi:10.1371/journal.pone.0016260)

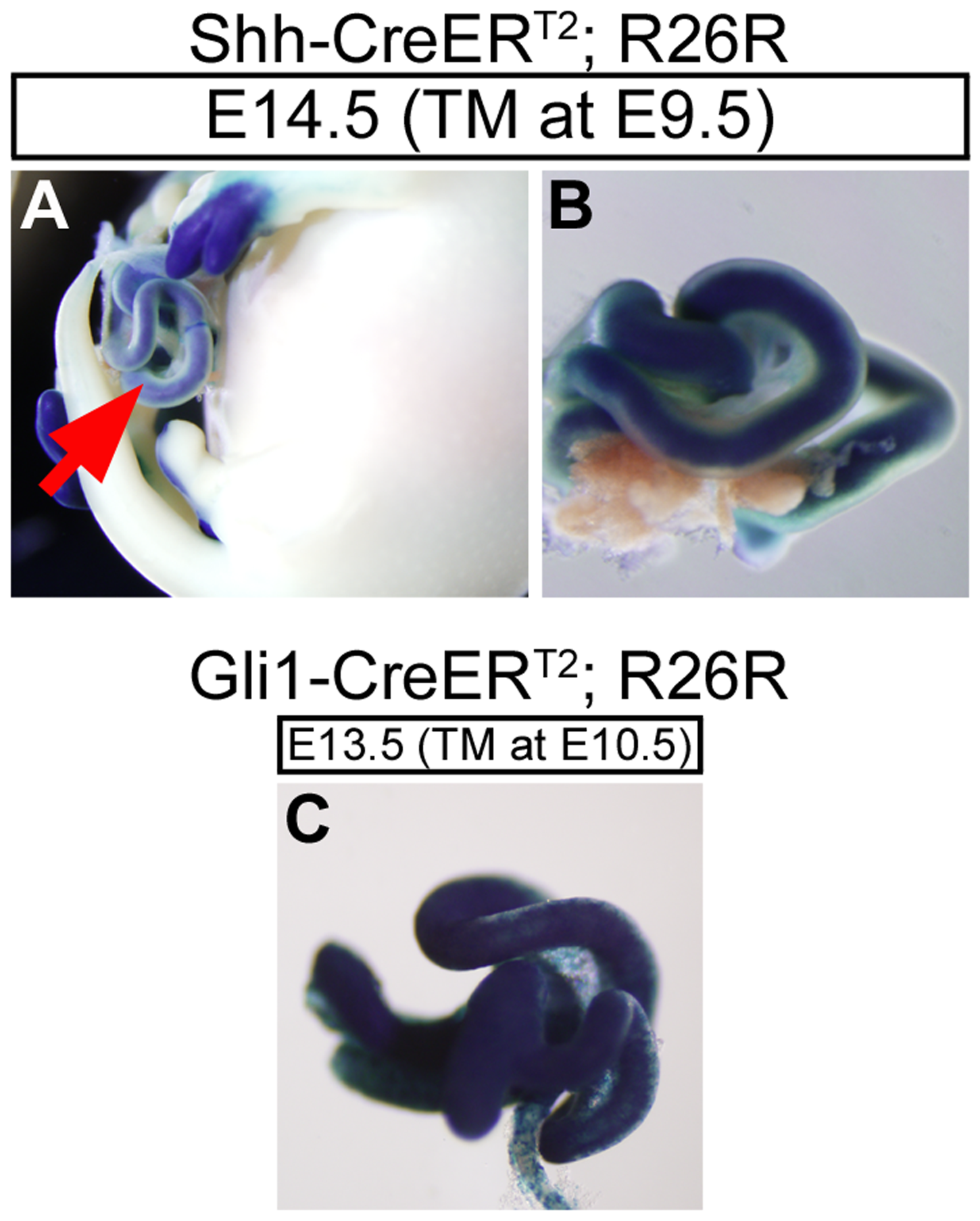

Supplement: Figure S1 — Cre recombinase activities of Shh-CreERT2 and Gli1-CreERT2in the developing gut. The Shh-CreERT2 activity was not observed in the ventral body wall at E14.5 upon tamoxifen treatment (4 mg/40 g maternal body weight) at E9.5 (A). Red arrow indicates the expression in the developing gut. The expression of Cre recombinase was detected in the endodermal epithelia of the midgut and posterior part of limb buds (B). The activity of Gli1-CreERT2 was also observed in the embryonic gut, including a part of the mesentery (C). The Gli1-CreERT2; R26R embryo was treated with 4 mg/40 g bw of tamoxifen at E10.5 and harvested at E13.5. (TIF) [file pone.0016260.s001.tif]

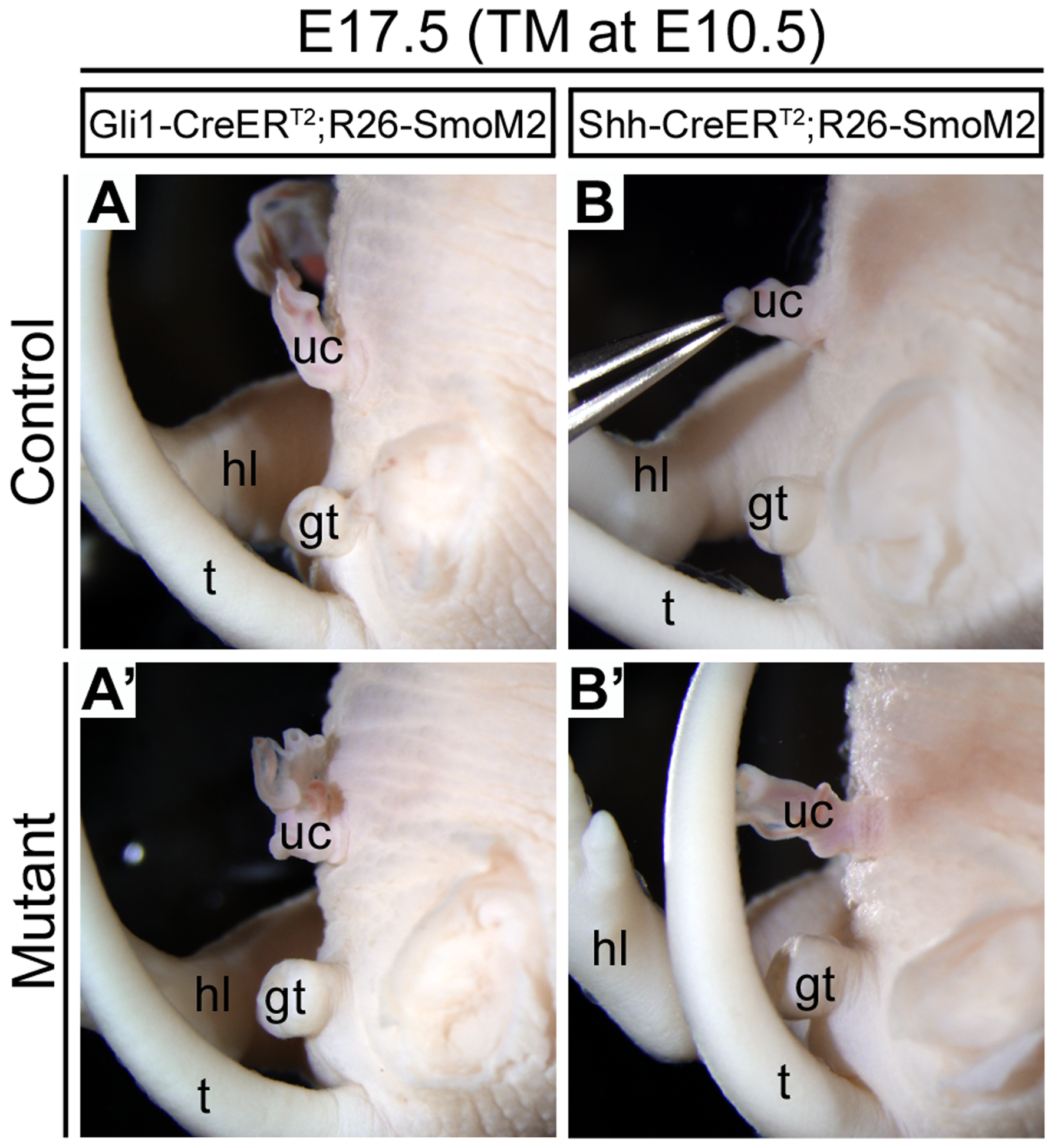

Supplement: Figure S2 — Augmentation of Hh signaling by utilizing Gli1-CreERT2 and Shh-CreERT2driver mouse lines. Both Gli1-CreERT2; R26-SmoM2 and Shh-CreERT2; R26-SmoM2 embryos did not display omphalocele phenotypes following administration of 4 mg/40 g bw of tamoxifen at E10.5 (A, A', B, B'). gt: genital tubercle, hl: hind limb, t: tail, uc: umbilical cord. (TIF) [file pone.0016260.s002.tif]

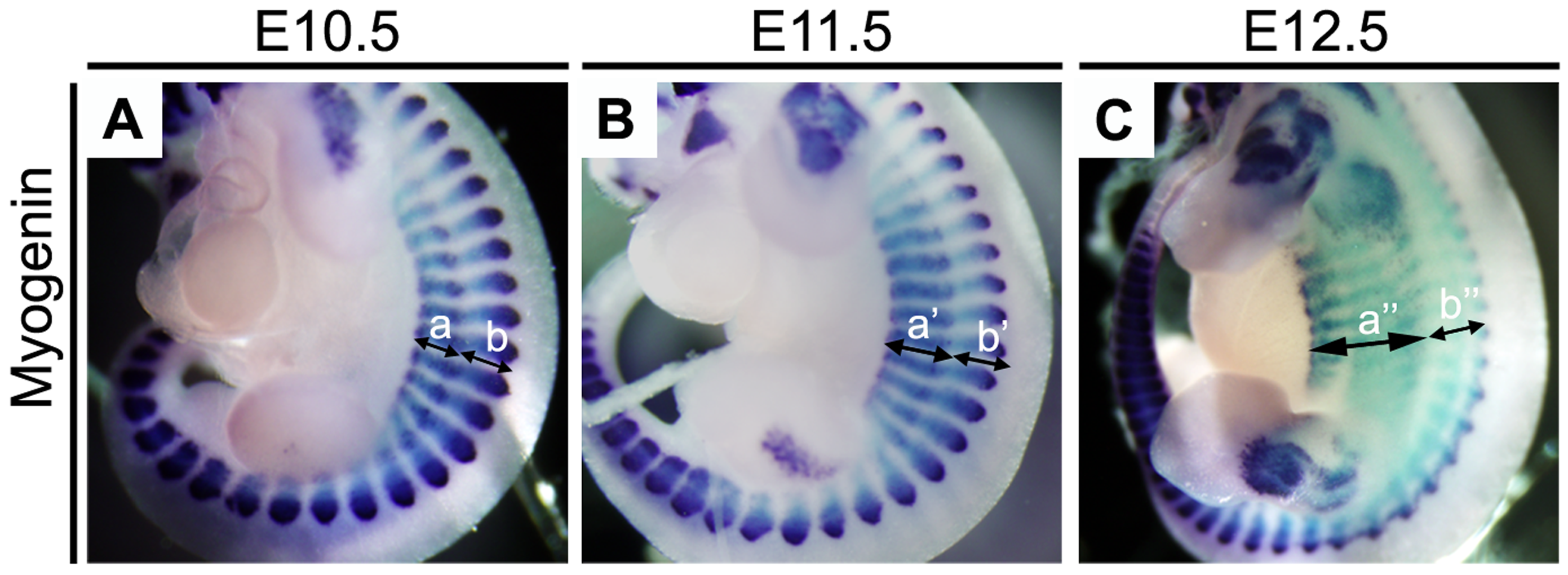

Supplement: Figure S3 — The expression of Myogenin in wild-type embryos at E10.5, E11.5 and E12.5. The ratio between primordia of hypaxial musculature (a, a' and a″) and epaxial musculature (b, b' and b″) was gradually increased during these stages, as hypaxial musculature (body wall muscle precursors) developed toward the midline (A-C). (TIF) [file pone.0016260.s003.tif]

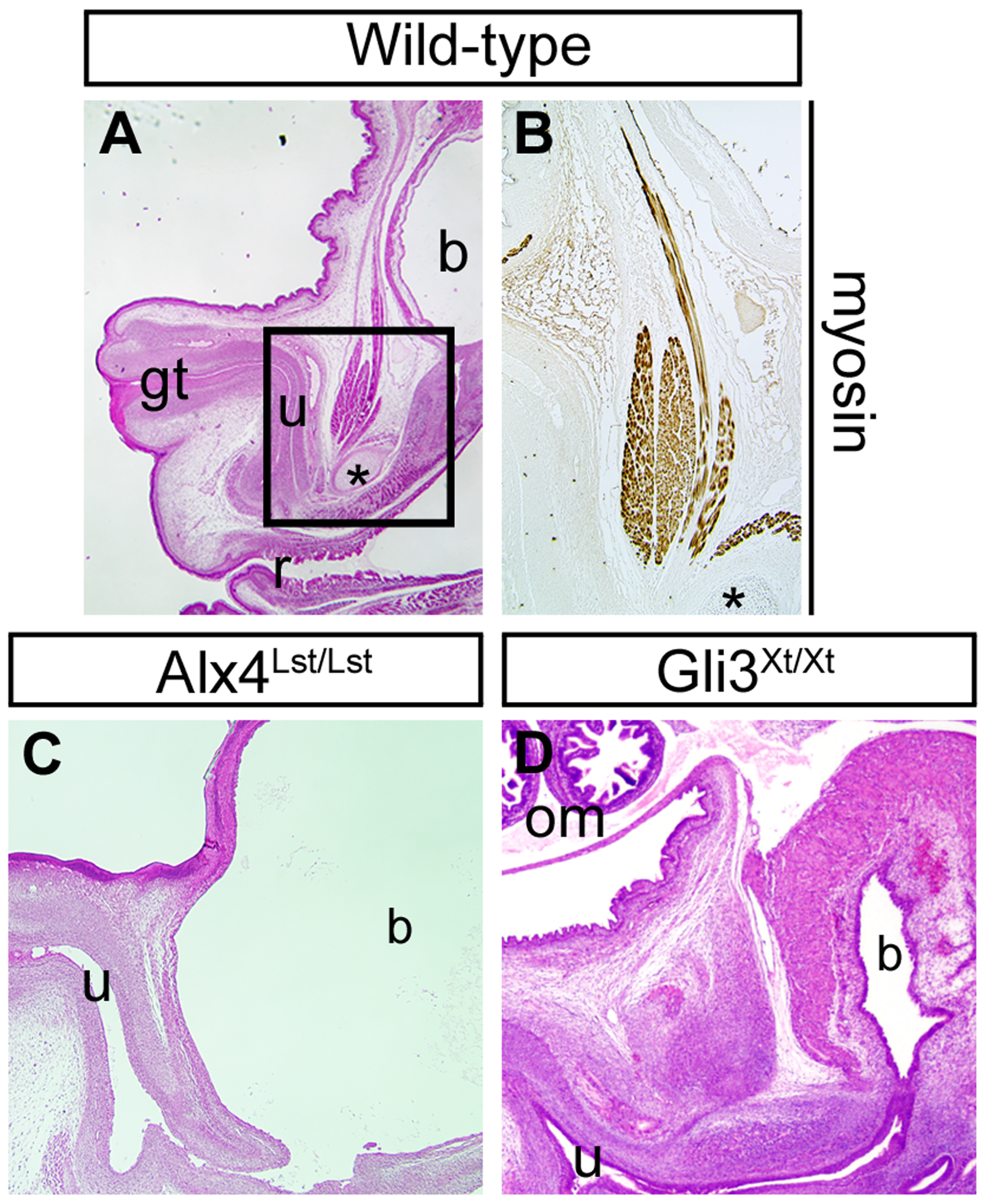

Supplement: Figure S4 — Absence of midline structures in Alx4Lst/Lst and Gli3Xt/Xt embryos. Sagittal sections of a control embryo at E18.5 displayed prominent pubic symphysis (A; asterisk) and abdominal muscle structures (B). Muscles were stained with Anti-Skeletal Myosin antibody (FAST) (Sigma). Neither Alx4Lst/Lst (C) nor Gli3Xt/Xt (D) mutant embryos developed pubic symphysis or abdominal muscles, as shown by sagittal sections. b: bladder, gt: genital tubercle, om: omphalocele, r: rectum, u: urethra. (TIF) [file pone.0016260.s004.tif]
